# COVID-19 outbreak and beyond: the information content of registered short-time workers for GDP now- and forecasting

**DOI:** 10.1186/s41937-020-00053-x

**Published:** 2020-09-11

**Authors:** Sylvia Kaufmann

**Affiliations:** Study Center Gerzensee, Foundation of the Swiss National Bank, Dorfstrasse 2, Gerzensee, 3115 Switzerland

**Keywords:** Bayesian analysis, COVID-19, Two-step regression, Forecasting, E23, E27, C32, C53

## Abstract

The number of short-time workers from January to April 2020 is used to now- and forecast quarterly GDP growth. We purge the monthly log level series from the systematic component to extract unexpected changes or shocks to log short-time workers. These monthly shocks are included in a univariate model for quarterly GDP growth to capture timely, current-quarter unexpected changes in growth dynamics. Included shocks additionally explain 24% in GDP growth variation. The model is able to forecast quite precisely the decrease in GDP during the financial crisis. It predicts a mean decline in GDP of 5.7% over the next two quarters. Without additional growth stimulus, the GDP level forecast remains persistently 4% lower in the long run. The uncertainty is large, as the 95% highest forecast density interval includes a decrease in GDP as large as 9%. A recovery to pre-crisis GDP level in 2021 lies only in the upper tail of the 95% highest forecast density interval.

## Introduction

The COVID-19 pandemic outbreak at the beginning of March 2020 has had negative effects on economic and social life in Switzerland as in other Western European countries unprecedented since World War II. For individuals, the lockdown mandated by the Swiss Federal Government on March 13 turned out less restrictive than in neighboring countries like France, Italy, and Austria, where people had to stay home and needed a permit to move. Nevertheless, economically and socially the imposed restrictions had a huge impact. A large share of the service sector, all personal services with physical contact, tourism, and all recreational and cultural businesses were shut down.

Without cash injections, the restrictions would have lead to business insolvencies and mass unemployment. To counteract the disruptive effects, the Federal Government in cooperation with Swiss banks installed a state-guaranteed loan scheme to provide enterprises with lifeline liquidity. Besides, a series of measures eased administrative procedures to apply for and obtain short-time work benefits and extended the group of workers eligible^1^. For example, the number of days in advance of the start of short-time work that employers have to apply (ten days) and the waiting period, i.e., the number of days in each settlement period that the employer has to cover short-time work benefits (two for the first six and three for following periods), were reduced to zero. The group of newly eligible workers included among others apprentices, persons employed on an hourly basis, and self-employed as well as employed managing staff. Particularly, no waiting time between the announcement, the start of short-time work, and the flow of benefits provided companies efficiently with emergency liquidity^2^.

**Fig. 1:**
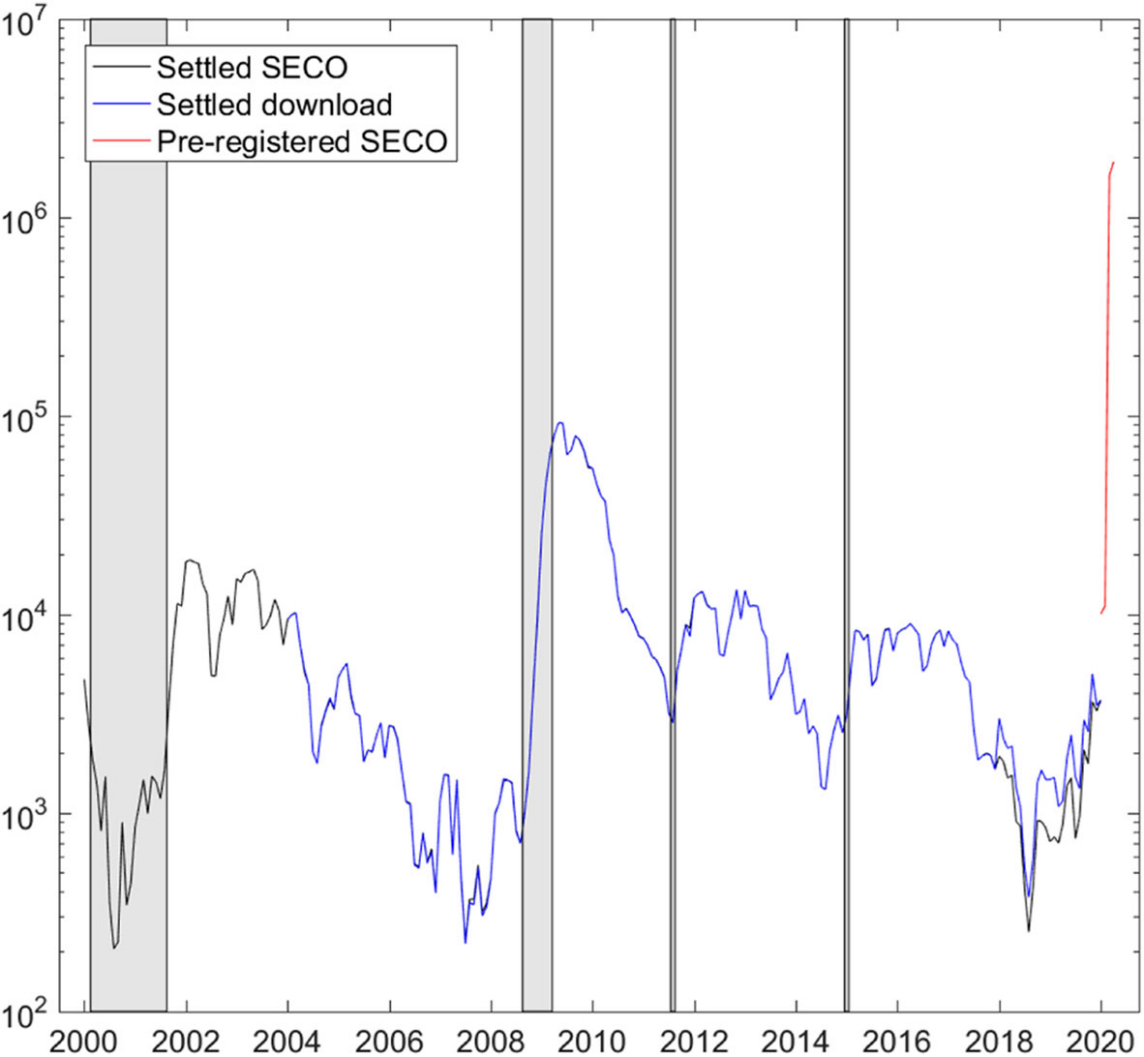
Short-time workers. Monthly frequency, logarithmic scale. Gray bars highlight the dotcom and financial crises, the introduction, and discontinuation of the euro-Swiss franc floor. Settled SECO: published pdf-file (https://www.seco.admin.ch/seco/de/home/Arbeit/Arbeitslosenversicherung/leistungen/ kurzarbeitsentschaedigung.html) as of May 1, 2020, January 2000–January 2020; Settled download: Download (https://www.amstat.ch/v2/index.jsp) as of May 2, 2020, January 2004–January 2020; pre-registered SECO: obtained by e-mail as of May 1, 2020, January 2020–April 2020

The seismic effects of the lockdown and the unbureaucratic procedure lead to a record-high, unprecedented increase in pre-registered short-time workers. In February 2020, roughly 11,000 persons were pre-registered for short-time work, a number slightly above the historical average of around 9000 (taking into consideration the high volatility in the series). In March, the number increased to above 1.6 million and reached more than 1.9 million in April. This corresponds to nearly 37% of employees. The incredible evolution is plotted in Fig. 1 on a logarithmic scale. This increase dwarfs the increase observed after the outbreak of the financial crisis at the end of 2008.

Obviously, short-time work directly impacts GDP. In real-time, the number of pre-registered short-time workers is available more immediately and at a higher frequency than a first estimate of quarterly GDP. Therefore, we will evaluate the information content of the number of registered short-time workers for GDP growth and form a first expectation for GDP prospects over the next one and a half years. The approach is simple and in the spirit of Romer and Romer (1989, 2004). In a first step, we extract unexplained variation or shocks in the log number of short-time workers. These shocks are included in an equation modeling GDP growth, and conditional on shocks observed ahead of GDP releases, we produce a forecast of GDP growth. There is no horse race in the paper, as the model is not intended to outperform other, more sophisticated forecasting models. Rather, the approach intends to illustrate that unusual indicators are useful to explore during periods of crisis, also to inform models built and specified during normal periods. The approach is Bayesian. Sequential updating will allow us to evaluate whether the number of registered short-time workers will stay informative as imposed measures will be abolished gradually in the course of the year.

The next section presents the data and introduces the econometric approach. Section 3 discusses the results. Section 4 concludes and provides an outlook.

## Data and econometric approach

### Data

Figure 1 plots the monthly series of short-time workers (number of employees) on a logarithmic scale and indicates the various sources. The linked, long data series starts in January 2000 and ends in April 2020. The data downloaded from www.amstat.ch (Settled download) starts in January 2004 and runs through January 2020. The series is augmented from January 2000 through December 2003 with observations published in a pdf-file (Settled SECO) on the website of the State Secretariat of Economic Affairs (SECO). I am grateful to Bernhard Weber from SECO, who provided the most recent numbers of workers pre-registered for short-time work from January 2020 to April 2020. The figure highlights in gray past crisis periods and critical monetary policy interventions: the dotcom and financial crises, the introduction, and the discontinuation of the euro-Swiss franc floor.

The figure illustrates the unprecedented increase in short-time workers during March and April. The number jumped from slightly above 11,000 workers pre-registered in January to 1.6 and 1.9 million in, respectively, March and April 2020, which represents nearly 37% of employees. The level exceeds the historical peak of roughly 90,000 short-time workers in the aftermath of the financial crisis by a factor of 21. Mainly two factors lead to this huge increase. To most effectively cut the COVID-19 infection chain, the Federal Government mandated the shutdown of a large share of the service sector, all personal services with physical contact, tourism, and all recreational and cultural businesses. To provide businesses with lifeline liquidity and counteract the disruptive effects that would otherwise lead to mass unemployment and business failures, the Federal Government substantially simplified administrative procedures and abolished the waiting periods for obtaining short-time work benefits. Specifically, the number of days in advance of the start of short-time work that employers have to apply for short-time work (usually ten days) and the number of days in each settlement period that employers have to cover employees’ short-time work benefits (usually two in the first six and three in following periods) were reduced to zero. In addition, the group of those eligible for short-time work benefits was enlarged to, among others, apprentices, workers employed on an hourly basis, self-employed, and employed managing staff. Overall, compared to measures taken during the financial crisis, administrative procedures were considerably more eased during the COVID-19 pandemic^3^.

**Fig. 2:**
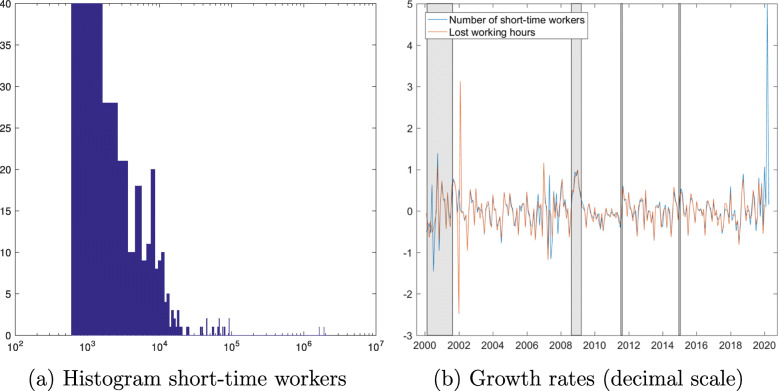
Short-time workers and lost working hours. Monthly frequency. Gray bars highlight the dotcom and financial crises, the introduction, and discontinuation of the euro-Swiss franc floor. Series merged from the three data sources (see footnote to Figure 1), the observation for short-time workers in January 2020 is taken from the e-mail source

Figure 2 shows the empirical distribution of short-time workers and plots growth rates in short-time workers and lost working hours. The histogram in Fig. 2a shows that the number of short-time workers has been fluctuating between 1000 and 10,000 most of the time. The cluster of observations below 100,000 refers to numbers recorded in the aftermath of the financial crisis. Excluding the numbers of pre-registered workers in 2020, the historical average has been slightly above 9,000. Figure 2b plots growth rates on a decimal scale, i.e., the first difference of the log level, of the number of persons working short-time and the number of lost working hours. Excluding again the pre-registered data for 2020, both series have a zero mean growth rate and their volatilities (one standard deviation) reach sizeable 0.39 (number of short-time workers) and 0.45 (lost working hours), i.e., 39% and 45%. The correlation between the level series is.97 and growth rates.69. We conclude that both series contain the same information and we could work with either of them. We choose the number of short-time workers in the following.

**Fig. 3:**
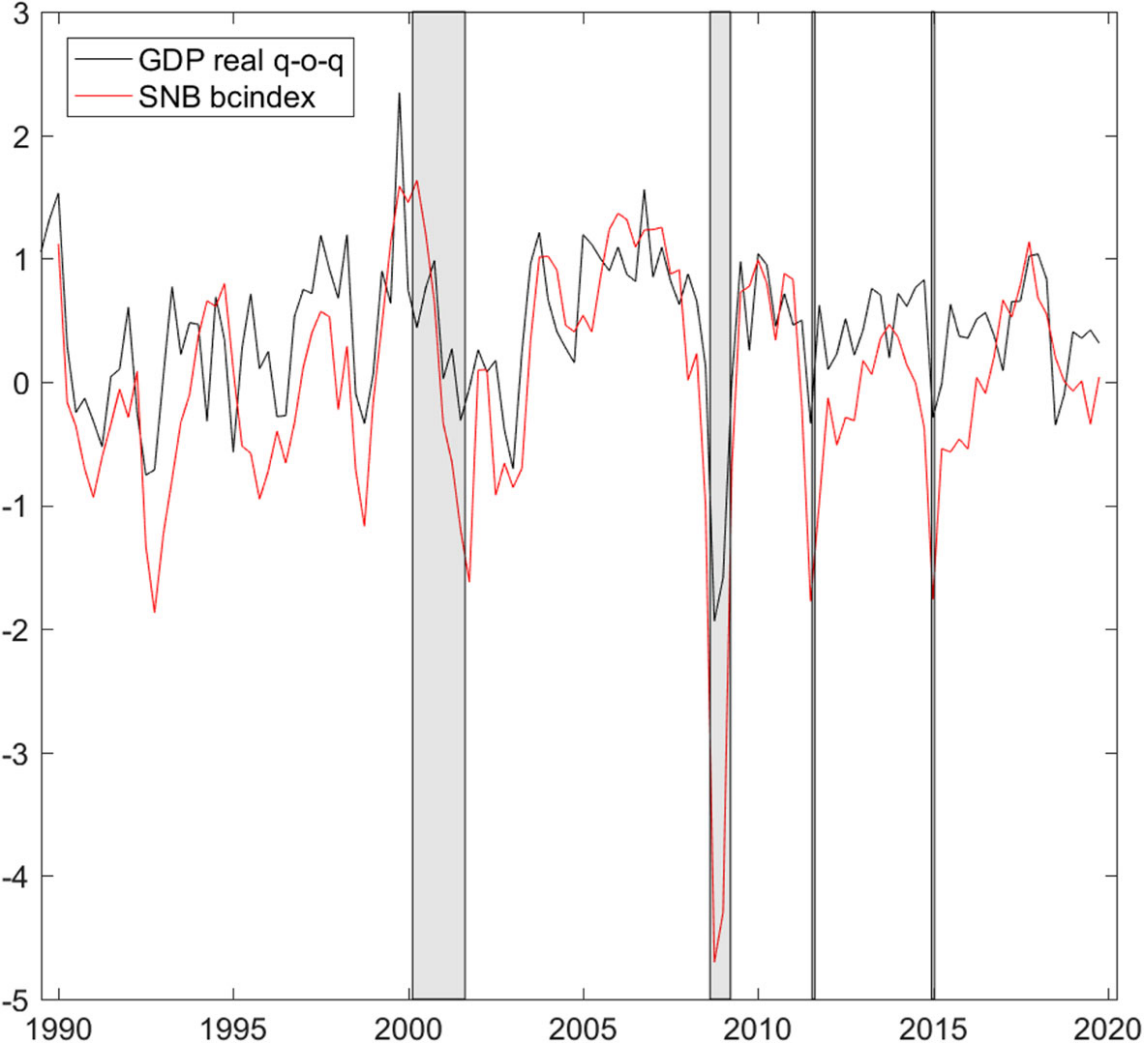
Growth rates (percentage scale). Real GDP (https://www.seco.admin.ch/seco/de/home/wirtschaftslage---wirtschaftspolitik/Wirtschaftslage/bip-quartalsschaetzungen-/daten.html, as of March 3, 2020), quarterly frequency, and SNB business cycle index SNB bcindex, (https://data.snb.ch/de/topics/snb#!/chart/snbbcich, as of April 21, 2020), quarter average of monthly frequency. Gray bars highlight the dotcom and financial crises, the introduction, and discontinuation of the euro-Swiss franc floor

Figure 3 plots on a percentage scale the series of interest, quarterly growth in real gross domestic product (GDP), as published on the SECO website. We plot along the quarter average of the monthly business cycle index produced and published by the Swiss National Bank (SNB). Given its considerable correlation (.77) with quarterly GDP growth, the index could serve as alternative and allow us to perform the analysis on a monthly basis. However, we observe that in particular since a couple of years before the financial crisis, the index apparently leads the decline in GDP growth. Therefore, we choose to work at the quarterly frequency to analyze and exploit the monthly information contained in the number of short-time workers for real GDP growth.

### Econometric approach

For the analysis, we take the logarithm of the number of short-time workers. Our goal is to form a short- to medium-term forecast of GDP growth (100 times the difference of the logarithmic level) at the end of the observation sample, including information extracted from variation in the number of short-time workers, which is not included already in past systematic GDP growth variation.

We have to address two issues. The first is the inherent endogeneity in historical, i.e., end-revised, time series. Even if the number of short-time workers is available at a higher frequency, the current-quarter monthly variation in this series is reflected in historical current-quarter GDP growth, and vice-versa. The second, less critical issue is to convert the information extracted at the monthly into lower-frequent quarterly information. To tackle the first one, we apply a procedure in the spirit of Romer and Romer (1989 and 2004) and extract first the unexplained variation or the shock in the (log) number of historical, or in-sample, short-time workers, $n^{s}_{t}$:

1 $$ n^{s}_{t}= c^{s}_{n} + \varphi_{1} n^{s}_{t-1} + \dots + \varphi_{l} n^{s}_{t-l} + \nu^{s}_{t}  $$

where the superscript *s* indicates the observations used to estimate the regression, $c^{s}_{n}$ is the intercept and $\nu ^{s}_{t}$ is i.i.d. *N*(0,*δ*^2^). Given that historical GDP growth figures reflect historical variations in the number of short-time workers, the lagged values $n^{s}_{t-j}$ in Eq. 1 purge current value $n^{s}_{t}$ from systematic variation also accounted for by lagged GDP growth. The residuals $\nu ^{s}_{t}$ reflect unexplained variation, i.e., the shock in $n^{s}_{t}$, that we include as additional information to model and forecast GDP growth.

There are various possibilities to use or convert the monthly shock series to match the quarterly frequency of GDP growth. We may use each first-, second-, or third-month shock, or add up shocks to cumulated quarterly information. We apply the latter approach and include within-quarter cumulated monthly shocks $\nu ^{s}_{qt}$ into the regression for quarterly GDP growth:

2 $$\begin{array}{*{20}l}  y^{s}_{t}=&c^{s}_{y}+ \theta_{0}\nu^{s}_{qt}+ \dots + \theta_{k}\nu^{s}_{q,t-k}+ \phi_{1} y^{s}_{t-1} + \dots + \phi_{p} y^{s}_{t-p} \\ &+ \sum_{j=1}^{3} \psi_{j}D_{jt}+\varepsilon^{s}_{t} \end{array} $$

where $c_{y}^{s}$ is the intercept and $\varepsilon _{t}^{s}$ are i.i.d. *N*(0,*σ*^2^). We allow shocks to have an effect up to *k* lags, *D*_*jt*_ is a set of quarterly dummy variables, *D*_*jt*_=1 if period *t* corresponds to quarter *j* and otherwise *D*_*jt*_=0.

We estimate (1)–(2) within a Bayesian framework; see the sampling steps described in the next subsection. Conditional on the model estimate, we draw impulse responses of GDP growth to a shock $\nu _{qt}^{s}$, for which highest posterior density intervals are available given the Bayesian approach. Forecasts and forecast distributions are available by a posterior predictive analysis, whereby we condition on shocks in the number of short-time workers available ahead of current-quarter GDP growth:

3 $$\begin{array}{*{20}l} y^{f}_{t}=&\hat{c}^{s}_{y}+ \hat{\theta}_{0}\nu^{f}_{qt}+ \dots + \hat{\theta}_{k}\nu^{f}_{q,t-k}+ \hat{\phi}_{1} y^{f}_{t-1} + \dots + \hat{\phi}_{p} y^{f}_{t-p}\\ & + \sum_{j=1}^{3} \hat{\psi}_{j}D_{jt} + \hat{\varepsilon}_{t} \end{array} $$

where $\nu ^{f}_{qt}$ represents within-quarter cumulated one-step ahead forecast errors (within-quarter cumulated differences between observed and forecast values, $n^{o}_{t}-n^{f}_{t}$) or in-sample within-quarter cumulated errors in case $\nu ^{f}_{qt}=\nu ^{s}_{qt}$. Likewise, in-sample values would substitute $y^{f}_{t-j}=y^{s}_{t-j}$. Longer-term forecasts condition on zero shocks $\nu _{qt}^{f}=0$, i.e., we assume that future, unobserved values $n^{o}_{t}$ correspond to forecasted values $n^{o}_{t}=n^{f}_{t}$. Equation 3 can be evaluated at the posterior mean of parameters $\hat {\beta }=E\left (\beta |\text {Data}\right)$. Evaluating for each draw $m=1,\dots, M$ out of the posterior, $\hat {\beta }=\beta ^{(m)}$, and imputing shocks for $\hat {\varepsilon }_{t}$, $\varepsilon _{t}^{(m)}\sim N(0, {\sigma ^{2}}^{(m)})$, we obtain draws from the forecast distribution of $y^{f}_{t}$.

Two points are worth discussing^4^. Equation 1 may also include lagged GDP growth if we think that lagged log short-time workers do not fully incorporate GDP growth dynamics, i.e., GDP growth may Granger-cause log short-time workers. The main justification for excluding lagged GDP growth is not the different frequencies of the series. The main justification is the timing of data releases. As for the current sample, data on log short-time workers are available up to April 2020, while GDP growth is available only up to the fourth quarter of 2019. If at all, quarterly GDP growth should enter (1) lagged by two quarters in order to match appropriately the historical release calendar. In the empirical analysis it turns out that the marginal effect of GDP growth lagged two quarters is significant, but negligible in terms of the scale of log short-time workers. Equation 2 sets out the stage for the effects of shocks in log short-time workers on GDP growth and the level of GDP. Discarding dummies *D*_*jt*_ for convenience and assuming *k*=0,*p*=1^5^, back-substitution yields

$$\begin{array}{@{}rcl@{}} y_{t}^{s} &=& c_{y}^{s} +\theta \nu^{s}_{qt} + \phi y_{t-1}^{s} +\varepsilon_{t}^{s}\\ &=& \sum_{j=0}^{t-1} \phi^{j} \left(c_{y}^{s} +\theta \nu_{q,t-j}^{s}+ \varepsilon_{t-j}\right)+\phi^{t} y_{0} \end{array} $$

If |*ϕ*|<0, the impact effect of one-time shocks $\theta \nu _{qt}^{s}$ or *ε*_*t*_ on GDP growth fades away over time at the rate *ϕ*, while the effect on the level of GDP accumulates in the long run, as Eq. 2 assumes a difference-stationary process for GDP. Deriving the Wold representation of (2) and using the Beveridge-Nelson decomposition (Beveridge and Nelson 1981), the long-run effect of a shock in $\nu _{qt}^{s}$ on the level of GDP is $\left (\theta _{0}+\dots +\theta _{k}\right)/\left (1-\phi _{1}-\dots -\phi _{p}\right)$. There is no permanent effect on the level of GDP if impact shocks to log short-time workers, $\theta _{0}\nu _{qt}^{s}$, are fully reversed over time, i.e., when $\theta _{0}+\dots +\theta _{k}=0$. The empirical analysis in fact shows that $\theta _{0}+\dots +\theta _{k}<0$, which suggests that shocks to log short-time workers have permanent effects on GDP similar to negative productivity shocks.

### Bayesian inference

Both Eqs. 1 and (2) can generically be represented in a regression matrix format

4 $$ Y=X\beta + \epsilon  $$

where *Y* represents the vector of *T* left-hand, in-sample observations, *X* is the regressor matrix with right-hand variables ordered in columns, and *ε*∼*N*(0,*κI*_*T*_), with *I*_*T*_ the identity matrix of dimension *T*, *κ*={*δ*^2^,*σ*^2^}.

We specify standard independent prior distribution for the parameters:

$$\pi\left({\beta} \right)= N\left(b_{0},B_{0}^{-1}\right), \quad \pi\left({\kappa} \right)=IG\left(g_{0},G_{0}\right)$$

where the normal prior for *β* is specified in terms of information *B*_0_ and *IG* represents the inverse gamma distribution.

Posterior inference on parameters combines the likelihood with prior information

5 $$ \pi\left({\beta,\kappa} | {Y,X} \right)\propto L\left({Y} |{X,\beta,\kappa} \right)\pi\left({\beta} \right)\pi\left({\kappa} \right)  $$

where the likelihood is $L\left ({Y} |{\cdot } \right)=\prod _{t=1}^{T} f(y_{t}|x_{t}, \beta,\kappa)$ with normal observation density *f*(*y*_*t*_|·)=*N*(*x*_*t*_*β*,*κ*), *x*_*t*_ row *t* of *X*.

To obtain a sample out of the posterior (5), we set initial values for *β* and *κ* and iterate over the following two steps:

1. Draw from *π*(*β*|*Y*,*X*,*κ*)=*N*(*b*,*B*^−1^),
  $$B=\kappa^{-1}X^{\prime} X + B_{0},\quad b=B^{-1}\left(\kappa^{-1}X^{\prime} Y + B_{0}b_{0}\right)$$
2. Draw from *π*(*κ*|*Y*,*X*,*β*)=*IG*(*g*,*G*),
  $$g=g_{0}+.5T,\quad G=G_{0}+.5\left(Y-X\beta\right)^{\prime} \left(Y-X\beta\right)$$

We discard a number of burn-in draws to remove the dependence from initial values and retain *M* draws for posterior analysis.

## Results

### The number of short-time workers

The long sample and the observed data variation allow us to specify relatively uninformative prior distributions and draw posterior inference mainly based on data information. We set *b*_0_=0, *B*_0_=0, *g*_0_=*G*_0_=1. This reduces prior information on regression parameters to zero (i.e., induces an infinite prior variance) and specifies a prior for the error variance where only the mode (0.5) and no moments exist. We iterate 3000 times over the sampler described in Section 2.3, discard the first 1000 and retain 2000 for posterior inference.

To decide on the lag length *l* in Eq. 1, we evaluate the Schwarz (BIC) and Akaike (AIC) information criteria based on the sample period January 2001–January 2020. Table 1 summarizes the results. As Fig. 1 does not reveal a strong seasonal pattern, we choose a lag length of *l*=3, which makes a compromise between BIC and AIC. Note that the main results are not sensitive to the choice of the lag length.

**Table 1:** Log number of short-time workers

*l*	12	6	4	3	2	1
BIC	− 1.82	− 1.84	− 1.88	− 1.91	− 1.93	− 1.93
AIC	− 2.02	− 1.94	− 1.96	− 1.97	− 1.97	− 1.96

Model choice: Schwarz (BIC) and Akaike (AIC) information criteria. Sample start: January 2001

We obtain the following posterior inference:

6 $$ \begin{aligned} \begin{array}{c} n^{s}_{t}= \\ {}\end{array} \begin{array}{c} 0.48\\ (0.15,0.83) \end{array} \begin{array} {c}+1.02 n^{s}_{t-1}\\ (0.90,1.17) \end{array} \begin{array}{c} +0.03 n^{s}_{t-2}\\ (-0.15,0.22) \end{array} \begin{array}{c} -0.11 n^{s}_{t-3}\\(-0.24,0.02) \end{array} \begin{array}{c} + e_{\nu t}\\ {}\end{array}\\ \begin{aligned} \sum_{j=1}^{3}\varphi_{j} = \\{} \end{aligned} \begin{array}{c} \quad0.94\quad,\\(0.90,0.98)\end{array}\ \begin{aligned}\delta^{2}= \\ {}\end{aligned} \begin{array}{c} \quad0.16\quad,\\ (0.13,0.18)\end{array}\ \begin{array}{c}R^{2}=0.91\\{}\end{array} \end{aligned}  $$

where the numbers indicate the posterior mean and the 95% highest posterior density interval (HPDI) in parenthesis. The sum of the coefficients indicates that the process is stationary and highly persistent. We explain a large share of data variance as indicated by an *R*^2^ of.91^6^.

**Fig. 4:**
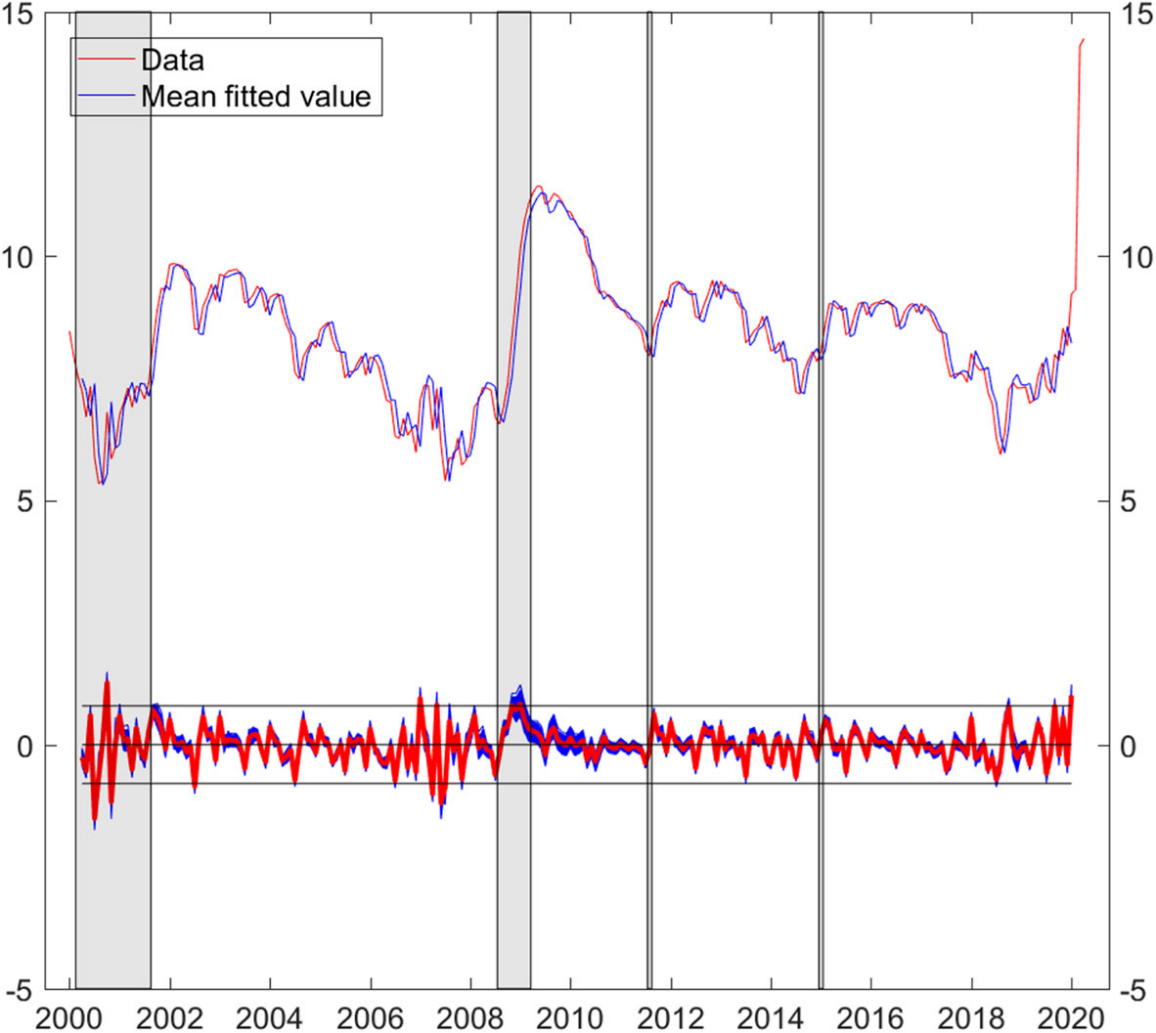
Log short-time workers. Data and mean fitted values, error (shock) series (mean in red). Sample period: April 2000–January 2020. Gray bars highlight the dotcom and financial crises, the introduction, and discontinuation of the euro-Swiss franc floor

Figure 4 plots the data along with the mean fitted values. All draws of error or shock series are plotted at the bottom of the graph, the mean is plotted in red, and the black lines indicate the interval of +/- two mean standard errors (0.8). We see the high volatility of shocks during the dotcom crisis and the persistent positive shocks during the financial crisis.

**Fig. 5:**
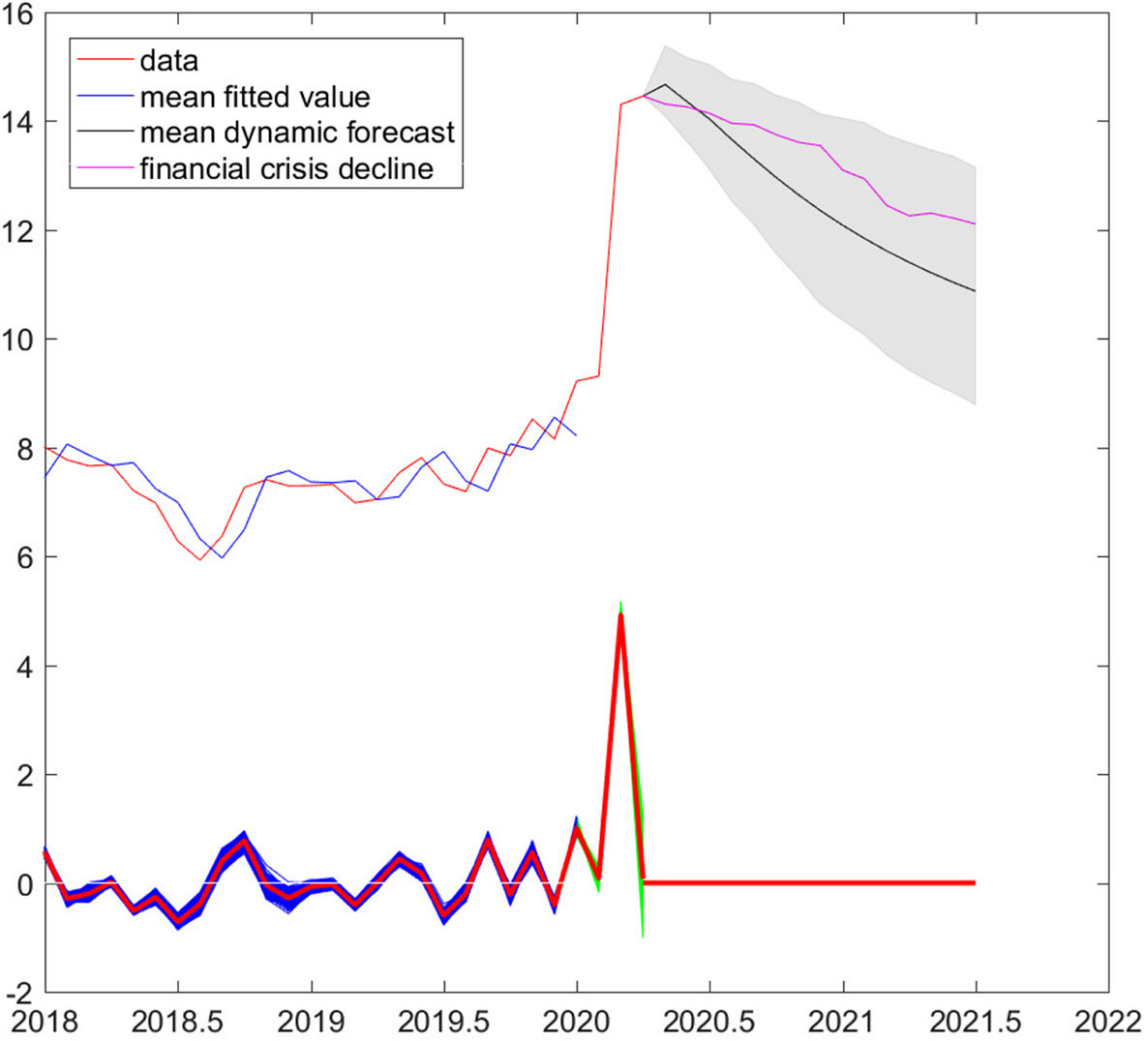
Log short-time workers, estimation sample: April 2000–January 2020. Top: Data and mean fitted values, mean dynamic forecast, and forecast proportional to the decline after August 2009 (financial crisis decline). Bottom: One-step ahead error (shock), in-sample (blue), out-of-sample (green), mean, and implied out-of-sample May 2020 to July 2021 (red)

In a second step, we extract model-implied shocks from log short-time workers observed from February to April 2020. Based on one-step ahead forecasts $n^{f(m)}_{t}$, we extract shocks $\nu _{t}^{f(m)}$:

7 $$\begin{array}{@{}rcl@{}} n^{f(m)}_{t}&=& c^{(m)}_{n} + \varphi^{(m)}_{1} n^{f}_{t-1} + \dots + \varphi_{l}^{(m)} n^{f}_{t-l} \\ \nu_{t}^{f(m)}&=& n^{o}_{t}-n^{f(m)}_{t}  \end{array} $$

where $n^{f}_{t-l}=n^{s}_{t-l}$ if the observation is part of the estimation sample and otherwise is one of the out-of-sample observations February to April. The superscript (*m*) indicates that we compute one-step ahead forecasts and forecast errors for each of the posterior draws. Equation 7 is also used to obtain dynamic forecasts from May 2020 onwards. Figure 5 plots in the upper part the data from 2018 onwards along with several forecasts for log short-time workers. The mean fitted values correspond to the in-sample mean one-step ahead forecasts, while the mean dynamic forecast (with shaded 95% highest forecast density interval) corresponds to out-of-sample dynamic forecasts based on (7). Despite the relatively high persistence estimate, the model predicts that the number of short-time workers would halve by August 2020 and decrease to roughly 52,000 in July 2021. Hence, the mean model-based forecast declines faster, i.e., represents a more optimistic scenario, than the forecast implied by a decline in short-time workers proportional to the decline observed from September 2009 onwards (financial crisis decline, see also Fig. 4). One-step ahead forecast errors or shocks are plotted in the lower part of the figure. In-sample shocks are plotted in blue, out-of-sample ones until April 2020 in green, and the mean in red. Obviously, the unprecedented increase in March leads to a huge shock, corresponding to an unexpected increase in short-time workers by a factor of roughly 148. The model accommodates quickly the new level, such that the further increase in April is attributed largely to the systematic part of the model. Correspondingly, the extracted shock for April is near 0. Setting shocks to zero from May 2020 onwards implies that future, unobserved log short-time workers follow the path predicted by the mean dynamic forecast.

### GDP growth

As for log short-time workers, we start out with uninformative priors and estimate Eq. 2 mainly based on data variation. We set *b*_0_=0 and *B*_0_=0, and for *σ*^2^, *g*_0_=*G*_0_=1. We iterate 4000 times over the sampler described in Section 2.3, discard the first 1000 and retain 3000 for posterior inference.

**Fig. 6:**
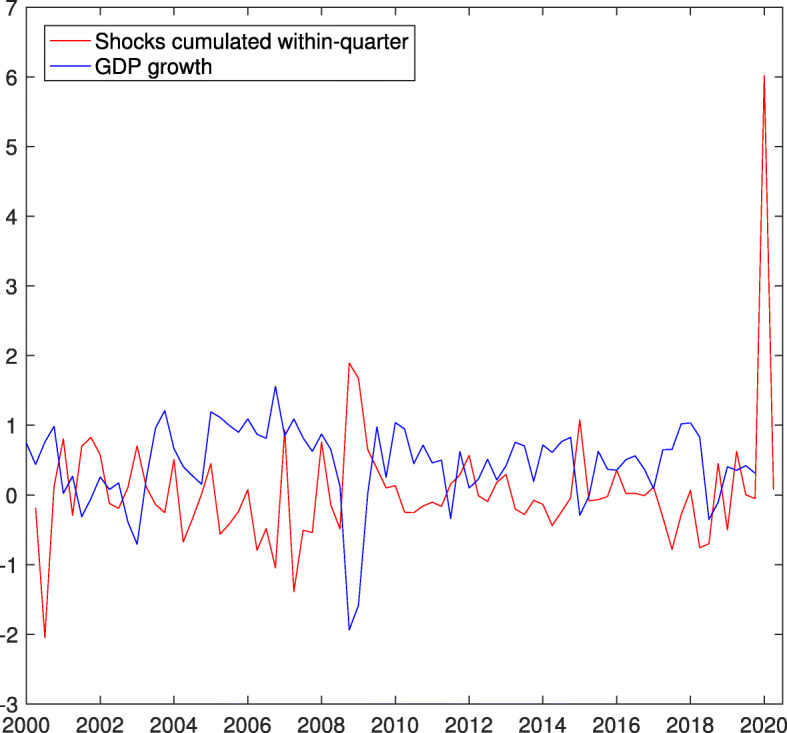
GDP growth and within-quarter cumulated shocks in log short-time workers. Sample period: Second quarter 2000–fourth quarter 2019

The shocks extracted from log short-time workers are cumulated within-quarter to match the frequency of GDP growth. Figure 6 plots the mean of cumulated shocks $\nu _{qt}^{s}$ along with GDP growth. The in-sample, negative correlation (−0.59) is substantial. We include the mean of cumulated shocks in Eq. 2. We specify the equation in the spirit of Romer and Romer (2004) and, as dealing with quarterly data, we set *k*=*p*=4 and include quarterly dummies. Posterior inference yields:

$${}\begin{aligned} \begin{array}{c} y^{s}_{t}=\\ {} \end{array} &\begin{array}{c} 0.50\\(0.18,0.83)\end{array}\begin{array}{c}-0.66\nu^{s}_{qt}\\ (-0.91,-0.39)\end{array} \begin{array}{c} -0.12\nu^{s}_{q,t-1}\\ (-0.43,0.19) \end{array}\\ &\begin{array}{c} -0.05\nu^{s}_{q,t-2}\\ (-0.37,0.26) \end{array} \begin{array}{c} -0.19\nu^{s}_{q,t-3} \\ (-0.44,0.08) \end{array} \\ &\begin{array}{c} +0.06\nu^{s}_{q,t-4}\\ (-0.21,0.34)\end{array} \begin{array}{c}+0.36y^{s}_{t-1}\\(0.05,0.65)\end{array} \begin{array}{c}-0.27y^{s}_{t-2}\\(-0.55,0.04)\end{array}\\ &\begin{array}{c}-0.21y^{s}_{t-3}\\(-0.49,0.07)\end{array} \begin{array}{c}-0.01y^{s}_{t-4}\\(-0.26,0.26)\end{array}\\ \end{aligned} $$

8 $$ {}\begin{aligned} &\begin{array}{c}+0.19D_{1t}\\(-0.19,0.61)\end{array} \begin{array}{c}-0.04D_{2t}\\(-0.45,0.39)\end{array} \begin{array}{c}-0.15D_{3t}\\(-0.49,0.25)\end{array} \begin{array}{c} +e_{\varepsilon t}\\ {~} \end{array}\\ \begin{aligned} \sum_{j=0}^{4}\theta_{j}=\\{} \end{aligned} &\begin{array}{c} \quad-0.97\quad,\\ (-1.61,-0.29)\end{array}\ \begin{aligned} \hspace{12pt} \sum_{j=1}^{4}\phi_{j}=\\{} \end{aligned}\begin{array}{c} \quad-0.14\quad,\\(-0.66,0.41)\end{array}\\ \begin{array}{c} \sigma^{2}=\\ {} \end{array} &\begin{array}{c} \quad0.19\quad,\\ (0.13,0.26)\end{array}\ \begin{array}{c} R^{2}=0.50\\ {} \end{array} \end{aligned}  $$

where numbers represent the posterior mean with the 95% HPDI in parenthesis. We explain overall 50% of data variation^7^. The information content of contemporaneous shocks is negative and highly significant. A unit shock in log short-time workers leads to a drop in current-quarter GDP growth of 0.7% and to a long-run decline of 0.8% in the level of GDP. As mentioned earlier (Section 2.2), this suggests that shocks to log short-time workers have long-term effects similar to negative productivity shocks. The results reveal that the equation is slightly over-specified. Some lags of shocks, some of the autoregressive lags, and dummy variables could be dropped. However, to capture as much systematic data variation as possible, we compute impulse responses and forecasts conditional on the full specification.

### Impulse responses and forecasts

Before further analyzing the model and computing a forecast, we may want to evaluate the model’s performance, in particular in predicting during crisis periods. Although we only have one observed crisis during the sample, we may use the financial crisis to confront the model with its forecasting ability. We use the same specifications, estimate both equations up to September or the third quarter of 2008, and forecast GDP out-of-sample after the outbreak of the financial crisis, conditional on shocks in log short-time workers observed through February 2009, i.e., the same number of observations available ahead of GDP as currently.

**Fig. 7:**
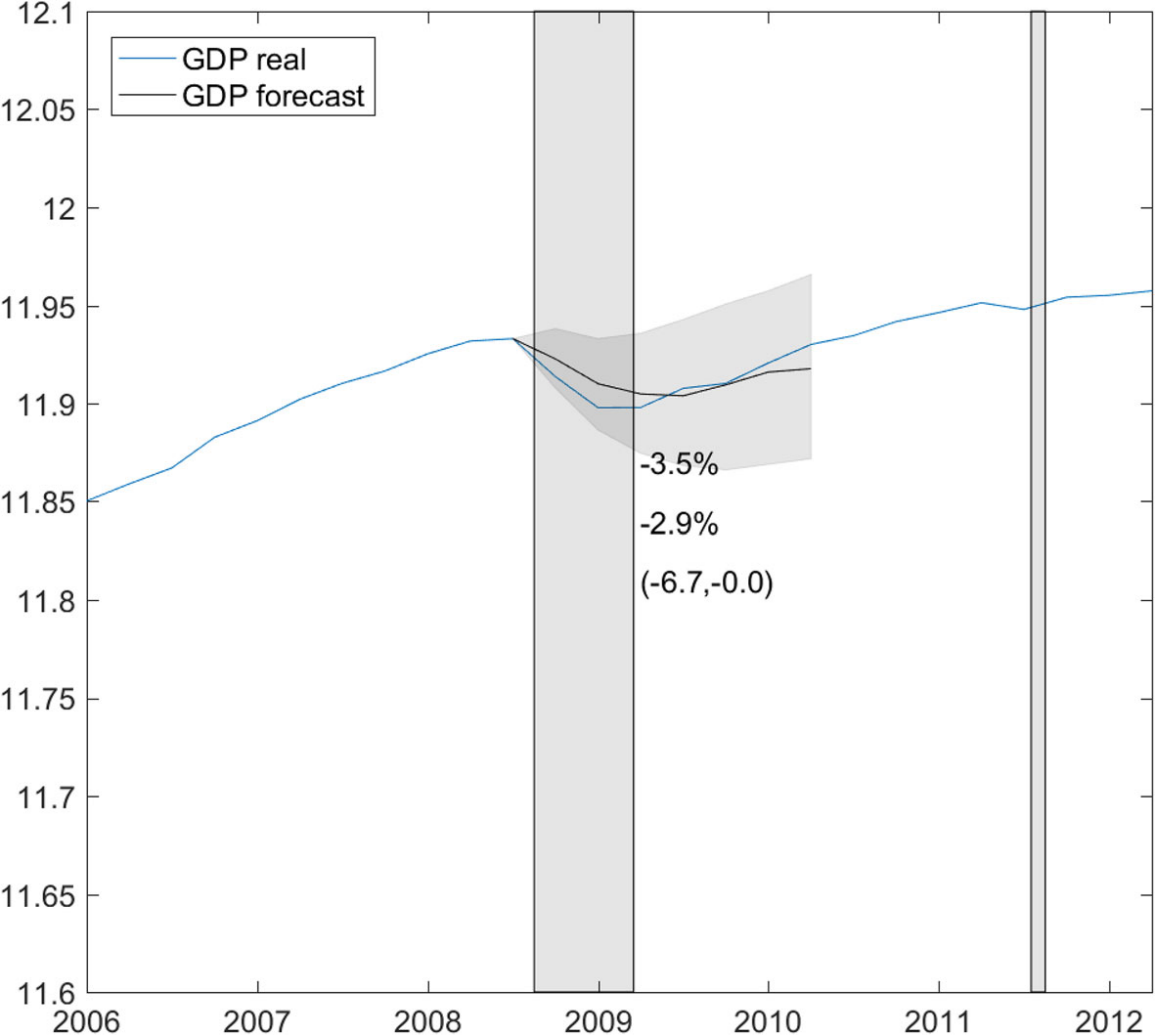
Out-of-sample GDP forecast, mean level forecast with 95% highest forecast density interval in gray. Sample period: second quarter 2001–third quarter 2008. Model estimated using as prior information posterior moments obtained for the long-sample estimate. Forecast period: fourth quarter 2008–third quarter 2010. Gray bars highlight the financial crisis and the introduction of the euro-Swiss franc floor

Given the shorter sample period, we specify informative prior distributions by using the posterior moments inferred from the long sample as prior moments in the short sample. Thus, we set $B_{0}=1/M\sum _{m=1}^{M} B^{(m)}$, $B_{0}=1/M\sum _{m=1}^{M} B^{(m)}$ and $G_{0}=1/M\sum _{m=1}^{M} G^{(m)}$, while *g*_0_=3 induces a loose shape for the error variance. The equations are thus estimated based on the information we accumulated by the beginning of 2020. Figure 7 plots the mean along with the 95% highest forecast density interval. The forecast declines less than what we observed, −2.9% compared with observed −3.5.%. Nevertheless, the 95% interval includes marginally observed GDP through the first quarter of 2009 and the mean level forecast is nearly on track with observed GDP from the second quarter 2009 onwards. Overall, the model fares well.

**Fig. 8:**
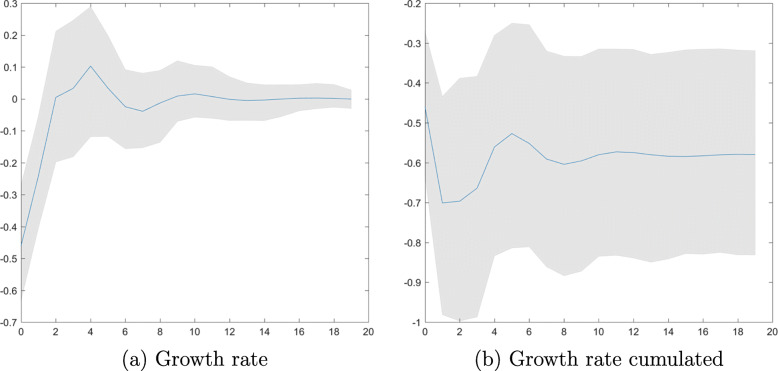
GDP growth. Impulse responses to a shock corresponding to an unexpected doubling of log short-time workers

We proceed by plotting impulse responses of GDP growth to a shock corresponding to an unexpected doubling of short-time workers. Figure 8a plots the impact drop in GDP growth of roughly 0.5%. Growth returns quickly to zero; there is no hump-shaped recovery. The drop in GDP is persistent, as reflected in Fig. 8b. The cumulated negative effect on GDP is around −0.6%.

**Fig. 9:**
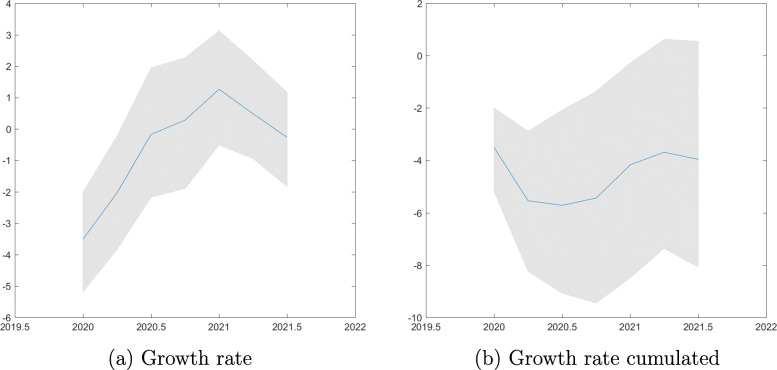
GDP growth. Forecast period: First quarter 2020–third quarter 2021. Forecast conditional on shocks in log short-time workers up to April 2020

**Fig. 10:**
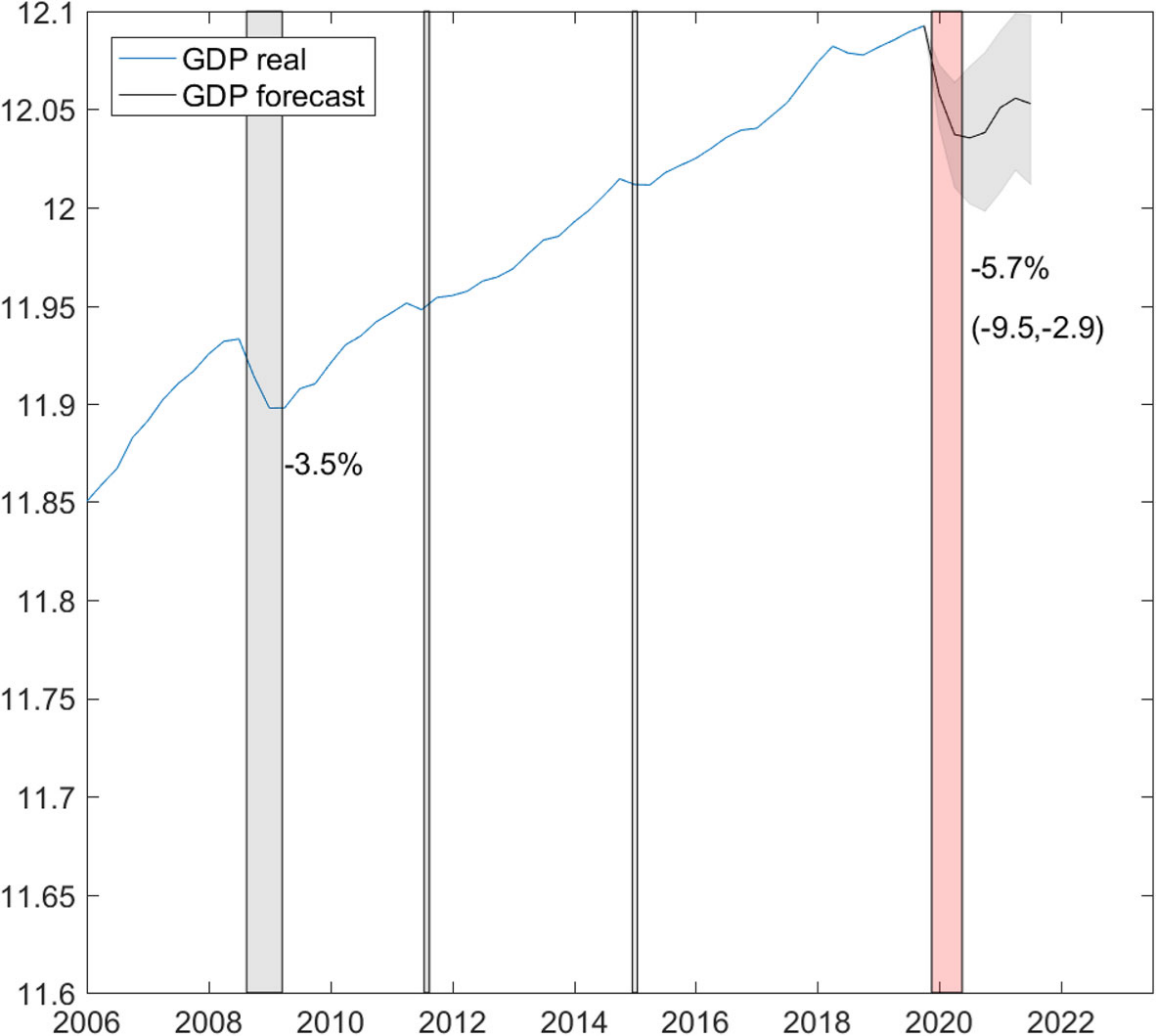
Out-of-sample GDP forecast. Sample period: second quarter 2001–fourth quarter 2019. Forecast period: first quarter 2020–third quarter 2021. Gray bars highlight the financial crises, the introduction, and discontinuation of the euro-Swiss franc floor; the red bar indicates the outbreak of the COVID-19 pandemic

Conditional on shocks in log short-time workers (see Fig. 5), we compute a forecast for GDP growth from the first quarter 2020 to the third quarter 2021. Figure 9 plots the mean forecast along with the 95% highest forecast density interval. The drop in GDP growth is 3.5% in the first quarter 2020 and GDP growth remains negative during the second quarter. There is a mild, hump-shaped recovery of about 1% forecasted for the first quarter 2021. Figure 9b shows the cumulated effects. The largest decline to −5.7% is reached in the third quarter 2020, while the 95% interval shows that the drop could be as large as 9%. Without any strong positive growth impulses, GDP will remain persistently 4% lower than pre-crisis in the long run. Figure 10 plots the data and forecast of log GDP. Compared with the financial crisis, the drop in GDP could be nearly twice as large this time^8^.

The mean level forecast is in line with the forecasts published by KOF Swiss Economic Institute at ETH on May 15 (−5.5%) and BAK Economics AG on May 7 (−5.3%) and 1% point higher than the forecast released by SECO on April 23 (−6.7%)^9^. All institutions forecast a rebound to pre-crisis GDP level for 2021. This level is included only in the upper tail of the 95% highest forecast density interval highlighted in Fig. 10.

## Conclusion

We exploit the information contained in the timely available number of persons registered for short-time work to obtain a now- and forecast of quarterly GDP growth. In a first univariate regression, we purge the log number of short-time workers from the systematic component, to obtain the shock or the unexpected variation in the series. The observed monthly shocks are cumulated to quarterly shocks and enter contemporaneously as well with lags in the second equation modeling GDP growth. The shocks explain 24% additional data variation. The model forecasts well the decrease in GDP during the financial crisis. The mean forecast for 2020 is in line with the forecasts published by forecast institutions in Switzerland. However, the recovery to pre-crisis level GDP forecasted by these institutions lies only in the upper tail of the 95% highest forecast density interval provided by the model.

The number of registered short-time workers appears to contain valuable information to now- and forecast GDP up to a horizon of one and a half years. As the crisis phases out and the economy smoothly accelerates towards the end of the year, the information content and the performance of the indicator may be re-evaluated.

## Data Availability

The datasets used and analyzed during the current study are available from the corresponding author on reasonable request.

## Abbreviations

AIC: Akaike information criterion
BIC: Schwarz information criterion
GDP: Gross domestic product
HPDI: Highest posterior density interval
SECO: State Secretariat of Economic Affairs
SNB: Swiss National Bank

## Footnotes

1 As in other countries providing short-time work benefit schemes, Swiss unemployment insurance subsidizes employees’ wages for reduced working hours caused by an unusual, temporary decline in demand. The scheme aims at encouraging firms to keep employees enrolled and avoid dismissal during these temporary periods of unusual low demand (Kopp and Siegenthaler 2017).

2 See Eichenauer and Sturm (2020) for an overview (in German) of economic policy measures taken to counteract the negative effects of the pandemic outbreak.

3 The interested reader may refer to the comprehensive study of Kopp and Siegenthaler (2017), who analyze the effects of short-time work benefits during and after the financial crisis, and view current changes in legal provisions decreed by the Federal Government (Bundesrat 2020), published in French, German, and Italian.

4 I thank the editor and an anonymous referee for raising corresponding questions.

5 The derivation encompasses (2), as an augmented distributed lag representation of order *k* and *p* may be represented in its companion form:

$$\boldsymbol{y}_{t}^{s}= \boldsymbol{c}_{y}^{s}+\Theta \boldsymbol{\nu}^{s}_{qt} + \Phi \boldsymbol{y}_{t-1} + \boldsymbol{\varepsilon}^{s}_{t}$$

with $\boldsymbol {y}_{t}^{s}=(y_{t}^{s},\dots, y_{t-(p-1)}^{s})^{\prime }$, $\boldsymbol {c}_{y}^{s}=(c_{y}^{s}, \mathbf {0}_{1\times p-1})^{\prime }$, $\boldsymbol {\nu }_{qt}^{s}=(\nu _{qt}^{s},\dots, \nu _{q,t-k}^{s})^{\prime }$, $\boldsymbol {\varepsilon }_{t}^{s}=(\varepsilon _{t}^{s}, \mathbf {0}_{1\times p-1})^{\prime }$ and

$$\Theta =\left[\begin{array}{l}\theta_{0},\dots,\theta_{k} \\ \mathbf{0}_{p-1 \times (k+1)}\end{array}\right], \ \Phi =\left[\begin{array}{l}\phi_{1},\dots,\phi_{p} \\ \mathbf{I}_{p-1}\ \mathbf{0}_{p-1 \times 1}\end{array}\right]$$

where **0**_·_, **I**_·_ denote, respectively, zero and identity matrices of respective dimensions.

6 When including GDP growth lagged two quarters as regressor, we obtain the following posterior inference (the coefficients on GDP growth are the same if we allow for month-specific effects of GDP growth lagged two quarters):

$$\begin{aligned} \begin{array}{c} {n^{s}_{t}=}\\ {~} \end{array} \begin{array}{c} 0.82\\ (0.41,1.25) \end{array} \begin{array}{c} +0.98 n^{s}_{t-1}\\ (0.84,1.11) \end{array} \begin{array}{c} +0.02 n^{s}_{t-2}\\ (-0.17,0.20) \end{array} \begin{array}{c} -0.09 n^{s}_{t-3}\\ (-0.22,0.04)\end{array} \begin{array}{c}-0.14 y^{s}_{q,t-2}\\(-0.24,-0.04)\end{array} \begin{array}{c} + e_{\nu t}\\ {~} \end{array}\\ \begin{aligned} \sum_{j=1}^{3}\varphi_{j}=\\{} \end{aligned} \begin{array}{c} \quad0.91\quad,\\(0.86,0.96)\end{array}\ \begin{aligned} \delta^{2}=\\{} \end{aligned}\begin{array}{c} \quad0.15\quad,\\ (0.13,0.18)\end{array}\ \begin{aligned} R^{2}= 0.91,\\{} \end{aligned} \begin{aligned} \textrm{BIC}=-1.92,\\{} \end{aligned} \begin{aligned} \textrm{AIC}=-1.99\\{} \end{aligned} \end{aligned} $$

The marginal effect of GDP growth lagged two quarters is significant. However, the additional explanatory power is negligible, less than half a percentage point, as explained variance (*R*^2^) does not increase and information criteria deteriorate (marginally). In terms of scale, the mean effect of average GDP growth ($0.14\bar {y}^{s}_{q}$) amounts to only 0.7% of average log short-time workers, $\bar {n^{s}}=8.31$.

7 When shocks to log short-time workers are excluded, the equation explains 26% of data variation.

8 When we condition on shocks extracted from log short-time workers declining in proportion to the decline observed from September 2009 onwards (financial crisis decline in Fig. 5), GDP drops by − 6.5% and the 95% highest forecast density interval is (−10.8, −2.8).

9 See KOF Swiss Economic Institute (2020), BAK Economics AG (2020), State Secretariat of Economic Affairs (SECO) (2020).
